# Development and validation of a heart failure with preserved ejection fraction cohort using electronic medical records

**DOI:** 10.1186/s12872-018-0866-5

**Published:** 2018-06-28

**Authors:** Yash R. Patel, Jeremy M. Robbins, Katherine E. Kurgansky, Tasnim Imran, Ariela R. Orkaby, Robert R. McLean, Yuk-Lam Ho, Kelly Cho, J. Michael Gaziano, Luc Djousse, David R. Gagnon, Jacob Joseph

**Affiliations:** 1 Massachusetts Veterans Epidemiology and Research Information Center (MAVERIC), Veterans Affairs Boston Healthcare System, Boston, MA USA; 2 Mount Sinai St Luke’s & Mount Sinai West Hospitals, New York, NY USA; 3 Division of Cardiology, Beth Israel Deaconess Medical Center, Harvard Medical School, Boston, MA USA; 4 Boston Medical Center, Boston University School of Medicine, Boston, MA USA; 5 Geriatric Research, Education and Clinical Center (GRECC), Veterans Affairs Boston Healthcare System, Boston, MA USA; 6 Institute for Aging Research, Hebrew SeniorLife, Boston, MA USA; 7 Department of Medicine, Beth Israel Deaconess Medical Center, Harvard Medical School, Boston, MA USA; 8 Department of Medicine, Brigham and Women’s Hospital and Harvard Medical School, Boston, MA USA; 9 Department of Biostatistics, Boston University School of Public Health, Boston, USA; 10 Cardiology Section, VA Boston Healthcare System, 1400 VFW Parkway, West Roxbury, MA 02132 USA

**Keywords:** Heart failure, Preserved ejection fraction, Electronic medical records, Epidemiology, Validation

## Abstract

**Background:**

Heart failure (HF) with preserved ejection fraction (HFpEF) comprises nearly half of prevalent HF, yet is challenging to curate in a large database of electronic medical records (EMR) since it requires both accurate HF diagnosis and left ventricular ejection fraction (EF) values to be consistently ≥50%.

**Methods:**

We used the national Veterans Affairs EMR to curate a cohort of HFpEF patients from 2002 to 2014. EF values were extracted from clinical documents utilizing natural language processing and an iterative approach was used to refine the algorithm for verification of clinical HFpEF. The final algorithm utilized the following inclusion criteria: any International Classification of Diseases-9 (ICD-9) code of HF (428.xx); all recorded EF ≥50%; and either B-type natriuretic peptide (BNP) or aminoterminal pro-BNP (NT-proBNP) values recorded OR diuretic use within one month of diagnosis of HF. Validation of the algorithm was performed by 3 independent reviewers doing manual chart review of 100 HFpEF cases and 100 controls.

**Results:**

We established a HFpEF cohort of 80,248 patients (out of a total 1,155,376 patients with the ICD-9 diagnosis of HF). Mean age was 72 years; 96% were males and 12% were African-Americans. Validation analysis of the HFpEF algorithm had a sensitivity of 88%, specificity of 96%, positive predictive value of 96%, and a negative predictive value of 87% to identify HFpEF cases.

**Conclusion:**

We developed a sensitive, highly specific algorithm for detecting HFpEF in a large national database. This approach may be applicable to other large EMR databases to identify HFpEF patients.

## Background

Heart failure with preserved ejection fraction (HFpEF) is increasing in prevalence [1], and already constitutes nearly 50% of patients with with clinical heart failure (HF) [2]. Despite the mounting public health burden, morbidity and mortality remain high because the exact pathophysiology of this disease is not well understood. Although multiple clinical trials have been conducted to examine the effects of phamacological therapies to improve the morbidity and mortality associated with HFpEF, spironolactone is the only specific therapy with a Class IIb (benefit = risk) indication for this condition [3, 4]. One of the major limitations in these clinical trials is the heterogeneity of HFpEF population in terms of clinical presentation and outcomes. Large electronic medical record (EMR) databases offer a rich resource for further research to advance the field of HFpEF; however, this requires curation of a well validated cohort. Current U.S. guidelines are broad in their definition of HFpEF, generally requiring a definite HF diagnosis with left ventricular ejection fraction (LVEF) of ≥ 50% [3]. However, asertaining this information from a large database is a major challenge. The Veterans Affairs (VA) is at the forefront of the use of an EMR, which has allowed the creation of large, centralized derivative databases of both administrative and clinical data. We hereby report on utilizing natural language processing (NLP) extraction of LVEF values and algorithm development and validation to derive an algorithm that is able to curate a cohort of HFpEF from a large national database.

## Methods

The study was approved by the VA Boston Healthcare System Human Subjects Subcommittee (IRB #2868). We used the national VA database to develop a HFpEF cohort utilizing EMR data recorded between 1/1/2002 to 12/31/2014. The VA uses Veterans Information Systems and Technology Architecture (VistA), which is a national integrated healthcare system with a comprehensive EMR across all the VA healthcare facilities. The VA has provided healthcare to millions of Veterans over the last two decades in more than 1700 hospitals, clinics, and nursing homes, with the majority of care recorded through VistA. A NLP tool which has been well established in VA database [5, 6], was used to extract EF values from the VA Text Integration Utilities (TIU) documents including history and physical examination notes, progress notes, discharge summary notes, echocardiography reports, nuclear medicine reports, cardiac catheterization reports, and other cardiology notes. The NLP application reported to have positive predicted values of 0.968 to 1.000 and sensitivities of 0.801 to 0.899 to extract EF values when tested across multiple data sources [6]. As published before [6], the following search terms were used to extract the ejection fraction (EF) values from TIU documents: “ventricle”,“ventricular”, “atrium”, “atria”, “echo”, “transthoracic”, “transesophageal”, “TTE”, “TEE”, “EF”, or “ejection” and “fraction”. Patterson et al. provided EF data which showed in the Veterans Aging Cohort Study, that the NLP system achieved F-scores of 0.872, 0.844, and 0.877 with precision of 0.936, 0.982, and 0. 969 to extract heart function measurements including ejection fraction from general clinic notes, echocardiogram reports, and radiology reports respectively [6].

**Fig. 1:**
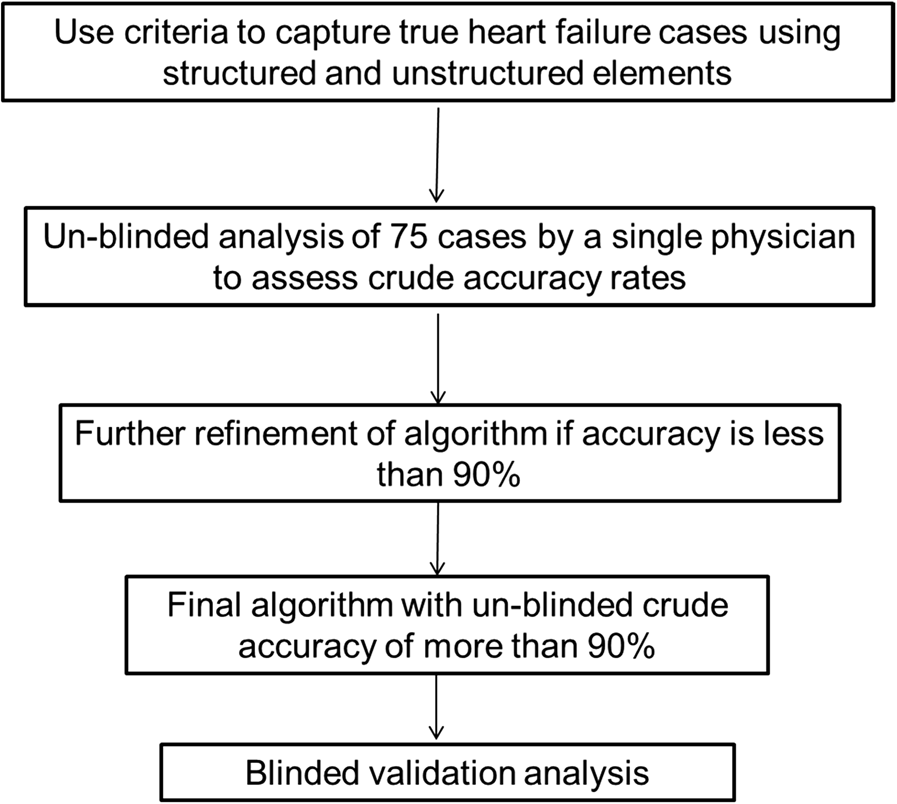
Algorithm development, refinement, and validation

HFpEF algorithm inclusion crtieria consisted of (1) *International Classification of Disease-Ninth Revision* (ICD-9) codes diagnosis of HF (any 428.xx), (2) either B-type natriuretic peptide (BNP) or aminoterminal pro-BNP (NT-proBNP) values recorded OR diuretic usage within one month of HF diagnosis, (3) availability of echocardiogram in our system, and (4) having all recorded EF values ≥50%. We excluded patients with constrictive pericarditis (ICD9 code 425.1) and hypertrophic cardiomyopathy (ICD9 code 423.2) and any previous history of HF. We specified the criteria of all EF values ≥50% to ensure a cohort of definite HFpEF through the period of study. As shown in fig. 1, there were 1,155,376 patients with any HF diagnosis from ICD9 code 428.xx. Refining the criteria for usage of a diuretic and elevation of BNP markers within 6 months of HF diagnosis resulted in 696,951 patients. The criteria were further refined for diuretic usage or BNP/pro BNP lab test within 1 month of HF diagnosis, as this might be representative of true recent HF diagnosis. Using data extracted from by the NLP tool, we then confirmed all recorded LVEF values ≥50% from TIU documents, leaving our cohort to be 98,709 patients.

**Fig. 2:**
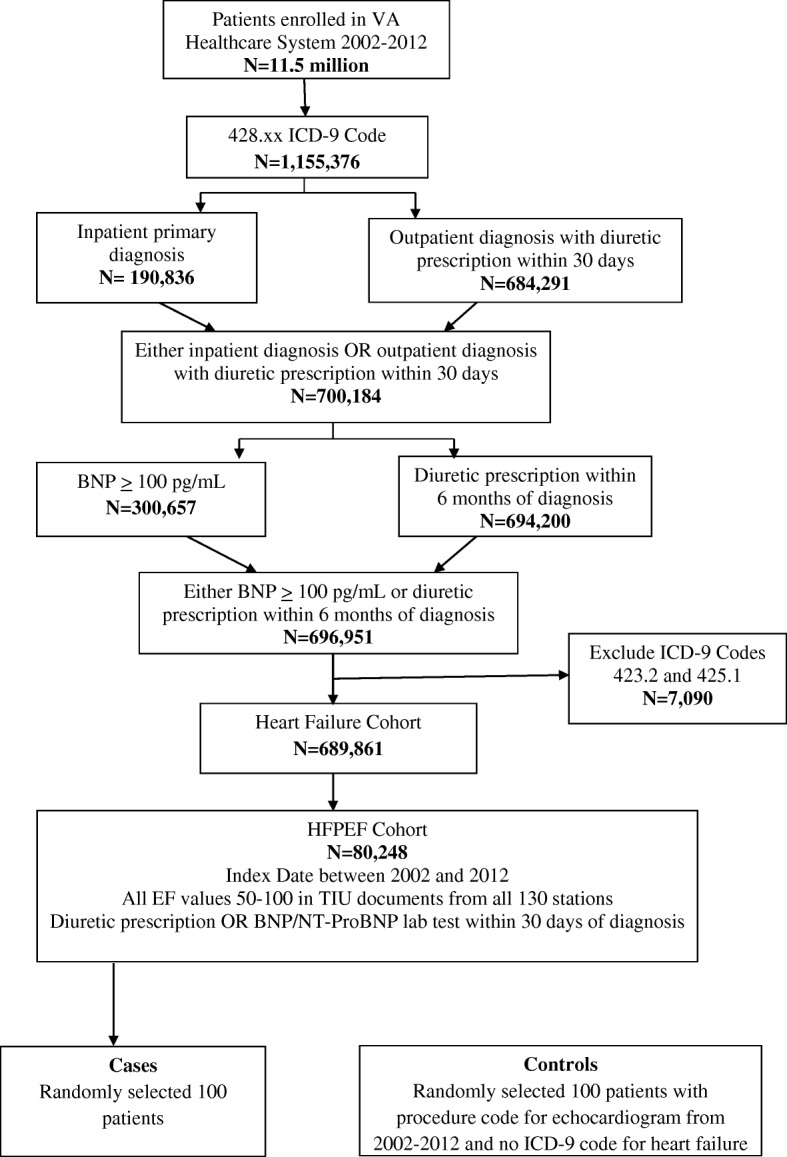
Algorithm developed to create a cohort of patietns with heart failure with preserved ejection fraction. *Abbreviations: VINCI, VA Informatics and Computing Infrastructure; ICD-9, International Classification of Diseases-9; BNP, B-type natriuretic peptide; HFpEF, heart failure with preserved ejection fraction; EF, ejection fraction; ProBNP, aminoterminal pro-BNP; TIU, Text Integration Utilities

Figure 1 shows the steps of the algorithm used for HFpEF development and refinement that led to validation. The final algorithm (Fig. 2) was designed using an interative process of designing a “draft” algorithm based on clinical and diagnostic criteria followed by rapid validation of a small number of charts and further refinement of the algorithm (Fig. 1). One independent physician (J.J.) initially validated 75 cases curated using the final algorithm to ensure that the algorithm was accurate enough for assessment by blinded validation.

We used clinical trials definition to validate definite and probable HF cases [7]. Definite HFpEF was defined as presence of at least one symptom, two signs, along with definite treatment of HF with diuretics/ other HF-specific therapies and all recorded LVEF values ≥50%. Probable HFpEF was defined as presence of one symptom and one sign, along with definite evidence of treatment of HF, or two signs along with definite evidence of treatment and all recorded LVEF values ≥50%. Likely HFpEF was defined as the presence of documentation of clinical diagnosis and continuing treatment of HF in physician notes but the absence of the documentation of the episode of HF with accompanying symptoms and signs likely due to the episode occuring in a medical facility outside the VA system, and all recorded LVEF values ≥50%. No HFpEF was defined as cases where there was no documentary evidence of HF.

Symptoms of HF included non-exertional and exertional dyspnea, orthopnea, paroxysmal nocturnal dyspnea, fatigue, decreased exercise capacity, and rapid increase in weight, edema, or abdominal girth. Signs included rapid weight loss on diuretics, elevation of jugular venous pressure, edema, elevation of BNP/NT-proBNP, pulmonary edema/rales, any radiologic evidence of HF (chest X-ray evidence of congestion or pleural effusion, ultrasound or CT evidence of pleural effusion), hemodynamic measurements showing elevated filling pressures, and presence of ascites or hepatomegaly.

Cases for chart review were randomly selected from the algorithm derived HFpEF cohort. Controls were randomly selected from a cohort who had a procedure code for echocardiogram in the medical records between 2002 and 2014 but no ICD-9 code for HF. Three independent reviewers (Y.P., T.I., and A.O.) completed the chart review of 100 cases and 100 controls in a blinded fashion, with each chart reviewed by two reviewers. Any discrepancies between the reviewers were addressed by further discussion and a consensus was achieved.

After manually reviewing the medical records for the 200 randomly selected patients, sensitivity, specificity, positive and negative predictive values were calculated to determine the ability of the algorithm to identify cases and controls relative to identification through manual chart review, which was considered the gold standard [8, 9]. Our final algorithm considered those manually classified as “likely HFpEF” as having a true diagnosis of HFpEF, but summary statistics were also calculated considering only “definite HFpEF” and both “definite HFpEF” and “probable HFpEF” as true HFpEF cases. All analyses were peformed using SAS Enterprise Guide 7.1 (SAS institute Inc., Cary, NC).

## Results

Comparison between algorithm-defined and manually reviewed cases and controls is shown in Table 1. The manual chart review confirmed 96/100 of cases and 87/100 of controls identified by our algorithm. Validation analysis (Table 1) showed that our algorithm had 88% sensitivity, 96% specificity, 96% positive predictive value (PPV), and 87% negative predictive value (NPV) to identify HFpEF cases from the EMR database. As mentioned in the Methods section, we included definite, probable, and likely HFpEF as true cases for our final algorithm. In our validation analysis, none of the cases validated belonged to the “no HFpEF” category. Interestingly 13 controls were identified as HFpEF; this may be secondary to the fact that our controls were derived from a cohort of subjects who had echocardiograms on file and thereby likely to have a diagnosis of HFpEF as a reason for performance of the echocardiogram. If only chart review defined “definite HFpEF” cases are included as true cases, the algorithm has 93% sensitivity, 74% specificity, 67% PPV, and 95% NPV. Finally, if chart review defined “definite” and “probable” HFpEF cases are considered true cases, the algorithm has 86% sensitivity, 78% specificity, 75% PPV, and 88% NPV.

**Table 1:** Comparison of 100 cases and 100 controls between algorithm-defined and manual chart review for HFpEF validation

			Algorithm		
			*Case*	*Control*	*Total*
Manual Chart Review (Gold Standard)	*Case*	Definite HFpEF	67	5	72
		Probable HFpEF	8	7	15
		Likely HFpEF	21	1	22
	*Control*	No HFpEF	4	87	91
	*Total*		100	100	200

*Abbreviations: *HF* heart failure, *HFpEF* heart failure with preserved ejection fraction

Table 2 demonstrates key demographics and clinical characteristics of our HFpEF cohort. Mean age was 72.5 ± 11.3 years. 96.5% were men and 12% were African-Americans. As would be expected, more than 50% had hypertension, and there was a high prevalence of atrial fibrillation, chronic obstructive pulmonary disease, chronic kidney disease, diabetes, peripheral vascular disease, and coronary artery disease. About two-thirds of the cohort were treated with renin-angiotensin system inhibitors, and 7.5% were on spironolactone.

**Table 2:** Characteristics of heart failure with preserved ejection fraction cohort

	HFpEF Cohort *N* = 80,248
Demographics	
Age (years)	72.5 (11.3)
Male (%)	96.5
Black or African American (%)	12.3
Body mass index (kg/m2) (%)	
Underweight (< 18.5)	1.5
Normal (18.5–24.9)	17.4
Overweight (25.0–29.9)	27.0
Obese (30.0–34.9)	23.7
Morbidly obese (≥ 35.0)	30.3
Clinical parameters	
Heart rate (beats per minute)	76.2 (16.0)
Systolic blood pressure (mmHg)	137.9 (23.4)
Diastolic blood pressure (mmHg)	73.0 (13.2)
Mean LVEF (%)	59.9 (5.9)
Laboratory parameters	
Serum sodium unit (mmol/L)	138.8 (3.7)
Hemoglobin (gm/dL)	12.4 (2.2)
BNP (pg/mL)	313.5 (252.2)
proBNP (pg/mL)	397.4 (292.6)
Serum creatinine (mg/dL)	1.4 (1.0)
eGFR (ml/min/1.73m^2^)	60.7 (24.9)
Serum potassium (mmol/L)	4.2 (0.5)
Serum BUN (mg/dL)	23.3 (10.5)
LDL cholesterol (mg/dL)	89.5 (35.2)
HDL cholesterol (mg/dL)	40.6 (14.0)
Triglycerides (mg/dL)	143.4 (99.5)
Total cholesterol (mg/dL)	157.4 (45.1)
Comorbidities	
Atrial fibrillation (%)	26.6
Stroke (%)	6.2
Hypertension (%)	88.4
Chronic liver disease (%)	5.0
Chronic obstructive pulmonary disease (%)	47.1
Chronic kidney disease (%)	26.4
Diabetes (%)	54.3
Hyperlipidemia (%)	66.3
Peripheral vascular disease (%)	27.6
Coronary artery disease (%)	45.5
Sleep apnea (%)	16.4
History of myocardial infarction (%)	8.4S
PCI/CABG (%)	5.2
Medications	
Angiotensin converting enzyme (%)	52.2
Angiotensin receptor blocker (%)	11.6
Beta blocker (%)	59.5
Calcium channel blocker (%)	40.3
Anticoagulants (%)	15.4
Antiplatelets (%)	11.6
Digoxin (%)	9.0
Loop diuretics (%)	69.9
Thiazide (%)	16.2
Statins (%)	53.9
Nitrates (%)	16.2
Spironolactone (%)	7.5

*Missing values: Race-5275; body mass index-6991; heart rate- 3681; Systolic blood pressure- 4259; Diastolic blood pressure-4259; Serum sodium-13,623; Serum potassium-13,192; Hemoglobin-33,786; Serum BUN-17,050; BNP-59,469; proBNP-76,896; Serum creatinine-14,085; eGFR-14,085; LDL cholesterol-47,133; HDL cholesterol-47,052; Triglycerides-46,795; Total cholesterol-45,165;

*Abbreviations: *HFpEF* heart failure with preserved ejection fraction, *LVEF* left ventricular ejection fraction, *BNP* B-type natriuretic peptide, *eGFR* estimated glomerular filtration rate

## Discussion

In a national cohort of VA patients, we demonstrated the feasibility of using NLP and EMR data to curate a HFpEF cohort with high specificity. Combining structured elements (i.e. ICD9 code, clinical characteristics) captured from EMR with a NLP tool to capture LVEF from unstructured data, our algorithm was able to rapidly and accurately curate HFpEF from a large EMR database. Our approach led to the creation of a cohort of veterans with HFpEF which allows for investigation of clinical predictors and outcomes in HFpEF.

Since HFpEF diagnosis requires clinical symptoms and signs inclusive of laboratory data and radiographic images and documentation of EF, correctly identifying HFpEF patients in a large cohort could be extremely challenging. This is a major limitation for population-based HFpEF research. The recent American College of Cardiology (ACC)/American Heart Association (AHA) guidelines suggested various cutpoints of EF values to diagnose different types of HF; including HFpEF with EF > 50%, heart failure with reduced ejection fraction (HFrEF) with EF < 40%, HF with intermediate EF with EF 40–50%, and HF with recovered EF whose values were < 40% in past but has now become > 50% [3]. To our knowledge, no previous HFpEF database has tried to validate HFpEF patients in a large database as per 2013 ACC/AHA guidelines thereby excluding people with HF with intermediate EF and HF with recovered EF. Hence our algorithm is able to predict HFpEF patients with reasonable accuracy while excluding patients with HF with intermediate EF and HF with recovered EF, due to our ability to extract all recorded LVEF values from EMR.

Similar approaches have been used to curate HF cohorts from EMR databases. For example, Bielinski and colleagues developed a tool to identify HF cases differentiated as HFpEF and HFrEF in an EMR database [10]. In this tool, HF was diagnosed using either ICD9 code of 428.0 or extracting the word “HF” from the problem list in clinical notes. NLP is a unique and novel tool that automatically extracts structured or semi-structured information from free text and has been used very commonly for clinical research [11]. Utilizing NLP to extract LVEF values from clinical documents and algorithms incorporating demographics, comorbidities, laboratory, and medications can assist better phenotyping of HFpEF in a large EMR database. In the above-mentioned study, HF was differentiated further into HFrEF and HFpEF using a NLP tool that extracted LVEF values from radiology reports. EF values were averaged from multiple measurements. In contrast to this, our algorithm also adds clinical characteristics such as usage of diuretics or an elevated BNP/NTproBNP value to ensure certitude of clinical heart failure. Furthermore, our algorithm ensured that all measured LVEF values were ≥ 50% which ensured that our cohort did not have any overlap with HFrEF or with the category of subjects who previously had lower LVEF or those who developed HFrEF during the course of follow-up in the database. Kottke et al. used a series of iterative algorithms to identify coronary and HF events from EMR [12] They used Intelligent Medical Objects, Inc. interface terminology (IMO terms) to classify coronary heart disease and HF, as major EMRs including Epic, Cerner, and NextGen incorporates IMO terms in theirs software. They showed that IMO, which is a more detailed coding system that tracks to ICD-9, ICD-10, and Systematized Nomenclature of Medicine – Clinical Terms along with troponin levels and echocardiograhy data, has near perfect agreement to classify cases with Cohen’s κ 0.99 (95%CI 0.98–1.00). Our database consists of Computerized Patient Record System software (universal EMR used in nationwide VA hospitals), which allowed us to uniformly extract variables from clinical documentations.

Similar to ours, other algorithms have been developed for various other clinical variables. Electronic Medical Records and Genomics (eMERGE) ePhenotyping algorithms were developed using a similar iterative approach, where each clinical disorder algorithm goes through a series of iterations until the algorithm performs to a desired criteria. These algorithms have been successfully utilized to define phenotypes in various clinical disorders including cataract [13], dementia [14], type 2 diabetes [15, 16], diabetic retinopathy [14], resistant hypertension [14], peripheral arterial disease [14], and primary hypothyroidism [17]. The eMERGE network have also developed automated phenotyping algorithms that can be deployed to rapidly identify diabetic and/or hypertensive chronic kidney disease cases and controls in EMRs using diagnostic codes, laboratory results, medication and blood pressure records, and textual information extracted from clinical notes, with positive predictive values of 96% and negative predictive values of 93% [18]. Adding NLP tools or some form of automated free-text narrative to extract data from clinical notes in EMR has also shown to improve the positive predictive value and sensitivity to accurately classify other medical comorbidities including psoriatric arthritis [19] and asthma [20]. The online natural language processing case finding algorithm was shown to be effective in identifying uncodified diabetes cases in Maine Health Information Exchange EMR database; indicating a strong potential for application of this method to achieve a more complete ascertainment of diagnosis of diabetes mellitus [15]. A multi-modal strategy like ours consisting of structured database querying, natural language processing on free-text documents, and optical character recognition on scanned clinical images, was also used to result in > 95% positive predictive value for identifying cataract cases [13]. The usage of medications and laboratory data used to treat and diagnose the clinical disorder respectively improves the positive predictive value, as shown in Chung et al. to identify rheumatoid arthritis in admininstrative and claim databases [21], in Levine et al. to identify skin and soft tissue infections in a primary care setting [22], and in Corey et al. to identify non alcoholic fatty liver disease disease in Research Patient Data Registry at Partners Healthcare [23].

Our cohort is similar to other published epidemiological and clinical trial cohorts like Treatment of Preserved Cardiac Function Heart Failure with an Aldosterone Antagonist (TOPCAT) [24], Organized Program to Initiate Lifesaving Treatment in Hospitalized Patients with Heart Failure (OPTIMIZE-HF) [25], and Acute Decompensated HEart Failure National REgistry (ADHERE) [26] cohorts, however few differences exists. Our VA cohort is predominantly male, and has more extensive comorbidity including a higher prevalence of hypertension and diabetes, in comparison to OPTIMIZE and ADHERE cohorts.

### Limitations and strengths

There were limitations in our algorithm. We may not have included all patients with HFpEF as we required stringent inclusion criteria, but this approach allowed us to more confidently identify true HFpEF patients. We did not have detailed echocardiographic parameters except EF values to characterize diastolic HF and its severity, although recent ACC/AHA guidelines [3] do not require these parameters in HFpEF classification. Care received outside of VA medical centers may not have been completely captured in this database, though the inclusion of data derived from Centers for Medicare & Medicaid Services enabled us to capture the vast majority of events in those over 65 years old. Events in veterans who were less than 65 years of age and ineligible for Medicaid could have been missed. Nonetheless, there were many strengths in this large cohort including a very refined HFpEF cohort with availability of clinical parameters to define HF and all patients having EF ≥ 50% at any time period during the follow up. We were able to exclude anyone with HFrEF, HF with intermediate EF, and HF with recovered EF. We included patients diagnosed with HF in ambulatory care setting; and as per Koudstaal et al. patients admitted to hospital with worsening HF but not known with HF in primary care have the worse prognosis and management compared to when they are known to have HF in an ambulatory care setting [27]. This will help us to phenotype HFpEF patients in real word setting; a problem which previous clinical trials failed to address.

## Conclusion

In conclusion, using a combination of structured, clinical parameters, and unstructured data, we have curated a well-refined algorithm to identify HFpEF patients to facilitate population-based research in HFpEF.

## Abbreviations

ACC: american college of cardiology
ADHERE: Acute Decompensated HEart Failure National Registry
AHA: american heart association
BNP: B-type natriuretic peptide
EF: ejection fraction
eMERGE: Electronic Medical Records and Genomics
EMR: electronic medical record
HF: heart failure
HFpEF: heart failure with preserved ejection fraction
HFrEF: heart failure with reduced ejection fraction
ICD-9: *International Classification of Disease-Ninth Revision*
IMO: Inc. interface terminology
IRB: institutional review board
LVEF: left ventricular ejection fraction
NLP: natural language processing
NPV: negative predicted value
NT-proBNP: aminoterminal pro-BNP
OPTIMISE-HF: Organized Program to Initiate Lifesaving Treatment in Hospitalized Patients with Heart Failure
PPV: positive predicted value
TIU: text integration utilities
TOPCAT: Treatment of Preserved Cardiac Function Heart Failure with an Aldosterone Antagonist
VA: veterans affairs
VistA: Veterans Information Systems and Technology Architecture
